# Human Serum Albumin Cys34 Adducts in Newborn Dried Blood Spots: Associations With Air Pollution Exposure During Pregnancy

**DOI:** 10.3389/fpubh.2021.730369

**Published:** 2021-12-23

**Authors:** William E. Funk, Nathan Montgomery, Yeunook Bae, Jiexi Chen, Ting Chow, Mayra P. Martinez, Fred Lurmann, Sandrah P. Eckel, Rob McConnell, Anny H. Xiang

**Affiliations:** ^1^Department of Preventive Medicine, Feinberg School of Medicine, Northwestern University, Evanston, IL, United States; ^2^Department of Research and Evaluation, Kaiser Permanente Southern California, Pasadena, CA, United States; ^3^Sonoma Technology, Inc., Petaluma, CA, United States; ^4^Department of Preventive Medicine, Keck School of Medicine, University of Southern California, Los Angeles, CA, United States

**Keywords:** adductomics, air pollution, newborn, dried blood spot, oxidative stress

## Abstract

**Background:** Increasing evidence suggests that exposure to air pollution during pregnancy is associated with adverse pregnancy outcomes. However, biomarkers associated with air pollution exposure are widely lacking and often transient. In addition, ascertaining biospecimens during pregnacy to assess the prenatal environment remains largely infeasible.

**Objectives:** To address these challenges, we investigated relationships between air pollution exposure during pregnancy and human serum albumin Cys^34^ (HSA-Cys^34^) adducts in newborn dried blood spots (DBS) samples, which captures an integration of perinatal exposures to small reactive molecules in circulating blood.

**Methods:** Newborn DBS were obtained from a state archive for a cohort of 120 children born at one Kaiser Permanente Southern California (KPSC) hospitals in 2007. These children were selected to maximize the range of residential air pollution exposure during the entire pregnancy to PM_2.5_, PM_10_, NO_2_, O_3_, based on monthly estimates interpolated from regulatory monitoring sites. HSA-Cys^34^ adducts were selected based on previously reported relationships with air pollution exposure and oxidative stress.

**Results:** Six adducts measured in newborn DBS samples were associated with air pollution exposures during pregnancy; these included direct oxidation products, adducts formed with small thiol compounds, and adducts formed with reactive aldehydes. Two general trends were identified: Exposure to air pollution late in pregnancy (i.e., in the last 30 days) was associated with increased oxidative stress, and exposure to air pollution earlier in pregnancy (i.e., not in the last 30 days) was associated with decreased oxidative stress around the time of birth.

**Discussion:** Air pollution exposure occurring during pregnancy can alter biology and leave measurable impacts on the developing infant captured in the newborn DBS adductome, which represents a promising tool for investigating adverse birth outcomes in population-based studies.

## Introduction

Periods of fetal development and early childhood have long been recognized as a critical window of vulnerability for exposure to a number of environmental chemicals (1, 2), and higher prenatal exposures have been associated with adverse postnatal effects for many common pollutants (1–3). Increasing evidence suggests that exposure to certain air pollutants during pregnancy may be associated with adverse pregnancy outcomes including preterm birth (4–7), low birth weight (4–8), intrauterine growth restriction (4, 9, 10), congenital anomalies (11, 12), and autism spectrum disorders (13–16). However, associations between air pollution exposures and adverse birth outcomes remain poorly understood due to the paucity of *in vivo* markers of effects. Because prospectively collected biospecimens during pregnancy and infancy are rarely available, investigators routinely rely on exposure assessment methods that provide estimates of residential ambient air pollution that do not account for infiltration, exposure away from the home, exercise that increases ventilation rate, and other determinants of personal dose (17). Consequently, in the newborn population there is a critical need for new biomarker approaches for investigating relationships with prenatal exposure to air pollution that may have significant long-term impact on offspring.

While the underlying biological mechanisms linking air pollution and adverse birth outcomes are complex and are not fully understood, oxidative stress and inflammation are thought to play a central role in air pollution toxicity through the generation of reactive oxygen species (ROS) (18–20), altered antioxidant defense, and disruptions in homeostatic processes (19–21). Exposure to ambient levels of NO_2_ and O_3_ gases and fine particles trigger a cascade of oxidative stress-related events, including inflammation (22, 23), carbonyl stress (22, 24, 25), mitochondrial injury (22–24, 26), and altered gene expression (22, 25, 26). *In vivo* experiments have demonstrated rapid oxidative stress responses after exposure to concentrated ambient PM_2.5_, with an almost doubling of ROS in the lungs and heart of rats (27). Chronic exposures to ambient levels of PM_2.5_ are also associated with increased lipid and protein oxidation in humans (28, 29), potentially due to the presence of a variety of transition metals and/or free radical components present in PM from atmospheric chemical reactions (30). Oxidative stress also activates transcription factors (e.g., factor-κB and activator protein-1), which upregulate the expression of cytokines, chemokines, and other proinflammatory mediators (31–35).

Despite their important role in air pollution toxicity, ROS and other reactive small molecules in circulating blood are transient and therefore cannot normally be measured *in vivo*. This has motivated the use of protein adducts as biomarkers for estimating exposures to reactive electrophilic chemicals (18–20, 36–39). Cys^34^ protein adducts in human serum albumin (HSA, the most abundant protein in plasma) reflects an integration of exposure to short-lived electrophiles over the life span of the protein (e.g., the residence time of HSA is about one month) (40–42). As a result, the HSA-Cys^34^ adductome provides a deep interrogation of environmental exposures to reactive electrophiles over relatively long time periods. This study focused specifically on the HSA-Cys^34^ loci, which acts as a highly efficient site for scavenging reactive chemicals from circulating blood including, direct oxidation products, modifications by small thiols, reactive aldehydes formed through lipid peroxidation, and a host of other reactive small molecules. While protein adducts have been used historically as biomarkers for estimating exposures to environmental toxicants (43–45), a large class of endogenously produced adducts have recently emerged as important biomarkers of environmental exposure that capture unique signatures of ROS, antioxidant capacity, and modifications to circulating thiols that can act as redox switches to regulate homeostatic processes that can be targeted in studies investigating links between prenatal environmental exposures and adverse health outcomes (18–20, 46–49).

In this study we applied a targeted adductomics approach to investigate relationships between air pollution exposures throughout pregnancy and HSA-Cys^34^ adducts measured in newborn dried blood spot (DBS) samples. Concentrations of PM_2.5_, PM_10_, NO_2_, and O_3_ were estimated with monthly averages during pregnancy based on the birth address. After isolating HSA from DBS samples and digesting the protein using trypsin, adducts were measured on the third largest tryptic peptide containing Cys34 using triple-quadrupole (QqQ) mass spectrometry. Adducts included in our panel were based on previous studies (18–20, 46–49) and included direct oxidation products, small thiol compounds related to antioxidant capacity, and markers of lipid peroxidation (Table 1). Direct oxidation products in our adduct panel include *S*-sulfinic acid (addition of two oxygen molecules) and S-sulfonic acid (addition of three oxygen molecules), which are directly formed through reactions with ROS. Adducts formed with small thiol compounds, including S-Cys, S-GSH, *S-*γ-GluCys, and *S*-CysGly, can serve as biomarkers of antioxidant defense. Because low-molecular-weight thiols serve as antioxidants in blood, they can be depleted during periods of increased oxidative stress. As a result, decreased concentrations of HSA-Cys34 mixed disulfides is indicative of increased oxidative stress (20). Finally, *S*-crotonaldehyde adducts was measured as a biomarker of lipid peroxidation. During periods of increased oxidative stress, ROS react with polyunsaturated fatty acids and generate reactive aldehyde species that can modify HSA-Cys34 through Michael additions (53).

**Table 1:** Adducts quantified in newborn DBS samples.

***m/z*, 3+, observed**	**Added mass (Da)**	**Retention time (min)**	**Putative annotation**	**Elemental composition**	**References**
796.43	−45.99	4.82	Cys34 → Gly	-CH_2_S	(20, 46–51)
811.76	1.01	5.22	unmodified T3	+H	(18–21, 46, 48–50, 50, 51)
816.43	15.02	5.32	methylation (not Cys^34^)	+CH_3_	(18–20, 46–50, 52)
822.42	32.99	4.40	*S*-sulfinic acid	+HO_2_	(18–21, 46–51)
827.09	47.00	4.95	*S-*Methylthiolation	+CH_3_S	(20, 46–48, 50, 51)
827.76	48.99	4.45	*S*-sulfonic acid	+HO_3_	(18–20, 20, 21, 46, 48–50)
830.77	58.03	5.05	*S*-methylisocyanate	+C_2_H_4_NO	(18, 46)
835.11	71.05	5.22	*S*-crotonaldehyde	+C_4_H_7_O	(18, 46–50)
841.43	90.00	4.88	S-Mercaptoacetamide	+C_2_H_4_NOS	(18, 46)
845.42	102.00	4.10	*S*-Cys (-H2O)	+C_3_H_4_NOS	(18, 20, 46–50)
851.43	120.01	3.50	*S*-Cys	+C_3_H_6_NO_2_S	(18–20, 46–48, 50, 51)
856.10	134.02	3.62	*S*-hCys	+C_4_H_8_NO_2_S	(18–20, 46–48, 50)
870.44	177.03	3.23	*S*-CysGly	+C_5_H_9_N_2_O_3_S	(18–20, 46–50, 52)
894.44	249.05	3.65	*S-γ*-GluCys	+C_8_H_13_N_2_O_5_S	(18–20, 46, 47, 50)
913.45	306.07	3.65	*S*-GSH	+C_10_H_16_N_3_O_6_S	(18–20, 46–50)

*Selected adducts in this study were annotated using high resolution mass spectrometry in previous studies*.

## Methods

### Study Design and Newborn DBS Samples

A cohort of 120 children born at a single Kaiser Permanente Southern California (KPSC) hospital in 2007 was identified. Children were selected based on estimated PM_2.5_ exposure during the entire pregnancy. Children with low and high exposures were oversampled to increase the variability of exposure and increase the power to detect associations (54). We obtained the newborn DBS samples collected at birth from these children from the California Biobank archive in the Department of Public Health. Children's sex and gestational weeks at delivery based on ultrasonography were obtained from KPSC electronic medical records databases. The Institutional Review Board from KPSC and the California Health and Human Services Agency approved this study and waived individual subject consent. A summary of cohort characteristics, air pollution exposure estimates, and adduct concentrations is provided in Table 2.

**Table 2:** Cohort characteristics, air pollution exposure, and adduct concentrations.

**Characteristic**	**Median (Q1, Q3) or *N* (%)**
**Cohort characteristics**
**Gender**
Boys	72 (60%)
Girls	48 (40%)
Gestational age at delivery (weeks)	39.0 (38.0, 40.0)
**Air pollutants**
**PM**_**2.5**_ **(ug/m**^**3**^**)**
Trimester 1	16.3 (10.4, 19.0)
Trimester 2	15.7 (10.7, 17.7)
Trimester 3	16.6 (10.6, 20.6)
Last 30 days of pregnancy	17.2 (10.2, 26.7)
**PM**_**10**_ **(ug/m**^**3**^**)**
Trimester 1	45.9 (34.9, 56.9)
Trimester 2	46.0 (34.2, 59.9)
Trimester 3	47.4 (34.1, 69.0)
Last 30 days of pregnancy	43.6 (31.8, 61.7)
**NO**_**2**_ **(ppb)**
Trimester 1	22.5 (20.9, 24.2)
Trimester 2	22.1 (20.6, 23.7)
Trimester 3	23.0 (17.9, 24.6)
Last 30 days of pregnancy	21.9 (17.8, 25.7)
**O**_**3**_ **(ppb)**
Trimester 1	51.2 (43.5, 60.9)
Trimester 2	59.2 (39.9, 70.4)
Trimester 3	45.6 (39.0, 57.0)
Last 30 days of pregnancy	40.0 (31.6, 60.0)
**Adducts (pmol/mg HSA)**
Cys^34^ F0E0 Gly	2.30 (1.72, 2.79)
unmodified T3	80.54 (49.91, 152.51)
methylation (not Cys^34^)	2.52 (1.48, 3.75)
*S*-sulfinic acid	5.48 (4.60, 6.50)
*S*-methylthiolation	0.27 (0.19, 0.45)
*S*-sulfonic acid	1.47 (1.11, 1.84)
*S*-methylisocyanate	0.50 (0.30, 0.75)
*S*-crotonaldehyde	1.38 (0.92, 1.80)
*S*-mercaptoacetamide	0.20 (0.11, 0.32)
*S*-Cys (-H_2_O)	0.84 (0.59, 1.05)
*S*-Cys	1.53 (0.95, 2.16)
*S*-hCys	0.13 (0.08, 0.23)
*S*-CysGly	0.31 (0.18, 0.40)
*S-γ*-GluCys	0.18 (0.12, 0.26)
*S*-GSH	15.65 (13.44, 18.61)

### Air Pollution Exposure Assessment

Average monthly air pollution exposure, including PM_2.5_, PM_10_, NO_2_, and O_3_ was estimated based on residential addresses at birth recorded in KPSC electronic medical record. The birth addresses were geocoded using MapMarker USA Version 28.0.0.11. Exposure data was compiled from the EPA regional air quality monitoring network across Southern California. To estimate the exposure at each residential location, we used the inverse distance-weighted monthly average from four closest monitoring stations within 50 km, except for geocoded locations within 0.25 km of a monitor, for which only data from the nearest monitoring station were used (55). Although the distance-weighted approach has limited accuracy in areas with sparse monitoring networks, performance is acceptable in Southern California due to the dense geographical network of historical measurements covering the region. In a previous Southern California study evaluating this method using leave-one-out validation for monthly monitoring station data, the coefficients of determination (R^2^) were 0.76, 0.73, 0.53, and 0.46 for O_3_, NO_2_, PM_2.5_ and PM_10_, respectively, with lower R^2^ values for PM attributed to the local (primary emission) dust component that is not regional (56). Bias was <1 ppb or 1 μg/m^3^. Each address was assigned the monthly average of the 24-h concentrations of PM_2.5_, PM_10_, and NO_2_. For O_3_, the monthly average of daily maximum 8-h concentrations was estimated. Averages of the monthly concentrations during each trimester and last 30 days of pregnancy were then aggregated from these monthly estimates, with each specific time window determined based on the last menstrual period (LMP) date estimated from fetal ultrasound used in clinical care. For months overlapping different exposure windows (e.g., first and second trimesters), the exposure was assigned proportional to the number of days in each window.

### Isolation of HSA From DBS Samples

HSA was isolated from DBS samples using the methods described by Yano et al. (48) with minor modifications. Briefly, 3.2 mm DBS punches were extracted in a 55 μL solution of 45% methanol in deionized water to precipitate hemoglobin and other interfering proteins. All solvents including deionized water were HPLC grade or higher. Samples were agitated at room temperature for 30 min and centrifuged at 20k RCF for 15 min at 4°C. The supernatant was diluted with 95 μL of a buffer consisting of 50 mM triethylammonium bicarbonate, 1 mM EDTA, with a pH of 8.0. 130 μL of filtered solution was then trypsin digested using 1 μL of 10 μg/μL trypsin. Protein digestion was performed using a Pressure Biosciences™ Barozyme HT48 with 30 pressure cycles of 20 kpsi for 50 s, then atmospheric pressure for 10 s for each cycle. Following pressure digestion, 3 μL of 10% formic acid was added to each sample to denature the trypsin and samples were centrifuged for 2 min at 10,000 × g. Analyses were then performed using 100 μL transferred to 300 μL borosilicate silanized glass vials (Microsolv Technology Corporation).

### Targeted Adductomics

A panel of adducts were chosen based on previous associations with air pollution exposure and oxidative stress-related processes, described in Table 1. Chromatographic separations were performed using an Agilent 1,260 Infinity system with an Agilent Poroshell 120, 3 × 50 mm column, with 2.7 μm diameter particles. An Agilent 1,260 Infinity Binary Pump A was used with a 10-min gradient, with solvents A: 0.1% v/v formic acid in deionized water; and B: 100% acetonitrile and the flow rate used was 0.7 ml/min.

The LC gradient was: 0–1 min 15% B, 1–3 min 30% B, 3–6 min linear gradient from 30 to 45% B, 6–8 min 100% B, 8–10 min 15% B. The column was maintained at 37? C using an Agilent Infinity 1,260 Thermostatted Column Compartment (G1316A). Samples were injected using an Agilent 1,260 Infinity MicroHP sample handling system, using 25 μl of sample per injection. To reduce sample carryover between runs, the needle was automatically rinsed for 10 s using 30% methanol prior to each injection.

Analyses were carried out on the Agilent 6,490 QqQ mass spectrometer using an iFunnel electrospray source with Jetstream technology. Mass spectrometer parameters were based on those used in Domanski et al. (57). Adduct levels were derived from the standard (not scheduled) SRM peak area of the [precursor (3+) –>y17 (2+)] transition for the principle peak coeluting with the [precursor (3+) –>b3+] qualifier peak (18). Collision energies were calculated using default parameters creation in Skyline (58) with no further optimization and the overall cycle time was ~750 ms. Other instrumental parameters were set using the Agilent Autotune functionality in positive ion mode, with Agilent ESI-L low concentration tuning mix (Catalog# G1969-85000) for mass calibration.

### Adducts Data Processing

Data acquired from targeted runs on the Agilent 6,490 QqQ was imported into a Skyline document containing the full set of transitions for each run. To quantitate the level of adducts across samples, the summed peak areas of 3 transitions per adduct species was divided by the combined peak areas of 3 housekeeping peptide transitions. The tryptic housekeeping peptide is adjacent in sequence to the T3 tryptic peptide and is used to estimate the concentration of HSA in each injected sample. Housekeeping peptide (Sequence: ^41^LVNEVTEFAK^50^) undergoes little enzymatic and non-enzymatic modification, is adjacent to the T3 peptide on HSA, and has similar tryptic digestion efficiency and ionization as the T3 peptide. As a result, this approach accounts for digestion and ionization efficiency across samples, as described in detail by Grigoryan et al. (18).

The limit of quantification (LOQ) for each adduct was calculated as 10 times the standard deviation of ion intensities from 46 blank samples (59). Blank samples were analyzed within each sample batch.

### Statistical Analyses

Descriptive statistics were used to examine cohort distribution. The distributions of adduct levels were not normally distributed and natural log transformation improved the distribution. The relationships between log of adduct levels and air pollution exposures were first examined by scatter plot followed by regression analysis. Data plots did not suggest strong non-linear associations and non-linear exposure terms in regression models were also largely not significant. Thus, linear regression models were used to explore associations between log of adduct levels (as dependent variable) and PM_2.5_, PM_10_, NO_2_, and O_3_ exposures adjusted for sex and gestational weeks at delivery. Additional adjustment for Hispanic ethnicity (46.7% of the cohort) did not affect the results. As this study was conducted as a proof of concept with limited sample size and limited previous knowledge about potential confounders, no adjustment for other factors was considered. All analysis was performed using SAS Enterprise Guide 7.1 (SAS Institute, Inc., Cary, NC). We assessed statistical significance based on a 2-sided *p*-value <0.05.

## Results

The 120 children included 72 boys and 48 girls, 46.7% with Hispanic ethnicity, who were delivered at a median of 39 weeks (interquartile range 38–40 weeks) of gestation. Table 2 presents the PM_2.5_, PM_10_, NO_2_ and O_3_ distribution for each trimester of pregnancy and last 30 days of pregnancy, as well as the distribution of the 15 adducts measured from DBS.

Regression results between air pollution exposure during pregnancy and each of the 15 individual protein adducts measured in newborn DBS samples are provided in Supplementary Table 1. Six different adducts were associated with the air pollution exposures, summarized in Table 3. The six adducts fall into three general classes of chemicals: direct oxidation products (*S*-sulfinic acid or dioxidation), small thiol compounds, including addition of glutathione to Cys^34^ (*S-*GSH), addition of cysteinylglycine to Cys^34^ (*S-*CysGly), and addition of γ-glutamylcysteine to Cys^34^ (*S-*γ-GluCys), addition of methanethiol to Cys^34^ (*S-*methanethiol), and addition of reactive aldehydes, namely addition of crotonaldehyde to Cys^34^ (*S-*crotonaldehyde). Results for each chemical class are summarized below.

**Table 3:** Significant associations between air pollution exposure on log-transformed adducts, adjusted for sex and gestational weeks at delivery.

**Adduct**	**PM**_**10**_				**PM**_**2.5**_				**O**_**3**_				**NO**_**2**_			
**Exposure period (Trimester or days)**	**1st**	**2nd**	**3rd**	**L30**	**1st**	**2nd**	**3rd**	**L30**	**1st**	**2nd**	**3rd**	**L30**	**1st**	**2nd**	**3rd**	**L30**
S-CysGly					↑
S-methylthiolation													↓
S-γ-GluCys								↑				↓
S-GSH											↑
S-sulfinic acid (dioxidation)		↑							↑		↓	↓			↑
S-crotonaldehyde	↓	↓			↓		↓*	↓	↓	↓*	↓*	↓*			↓	↓

*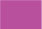 Antioxidant capacity*.

*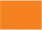 Direct oxidation*.

*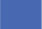 Lipid peroxidation*.

*Upward arrows represent positive associations and downward arrows represent negative associations*.

*P-values with p < 0.01 are in green, 0.01 < = p <0.05 are in red*.

P-values with p <0.0071 have *next to arrow (Bonferroni Correction: 0.05/7components to take care of multiple biomarker comparisons).

### Direct Oxidation Products

*S-*sulfinic acid was associated with air pollution exposure during pregnancy. Possitive associations were observed between *S-*sulfinic acid and PM_10_ exposure in the second trimester, *S-*sulfinic acid and O_3_ exposure in the first trimester, and *S-*sulfinic acid and NO_2_ in the third trimester [mean difference (MD), representing the change in the log of adduct associated with change in one unit of air pollution = 0.0032, 0.0043, 0.0146]. In contrast, negative associations were observed between *S-*sulfinic acid and O_3_ in the 3rd trimester and with O_3_ exposure in the last 30 days of pregnacy (MD = −0.0049, −0.0037).

### Small Thiol Compounds

*S-*GSH adducts were positively associated with exposure to O_3_ during the 3rd trimester (MD = 0.0035). In addition, significant associations were observed with *S-*CysGly and *S-*γ-GluCys adducts, which are two-amino acid precursors of GSH. *S-*CysGly was positively associated with PM_2.5_ exposure in the 1st trimester and *S-*γ-GluCys was positively associated with PM_2.5_ exposure duiring the last 30 days of pregnancy (MD = 0.0353, 0.0202). In contract, *S-*γ-GluCys was negatively associated with O_3_ exposure duiring the last 30 days of pregnancy (MD = −0.0131). Finally, *S-*methanethiol was negatively associated with NO2 exposure during the 1st trimester (MD = −0.0439).

### Reactive Aldehydes

Significant relationships were also observed between *S-*crotonaldehyde and air pollution constituients thoughout all trimesters of pregnacy. While strong positive assocaitions were observed between *S-*crotonaldehyde and O_3_ during the 3^rd^ trimester and last 30 days of pregnancy (MD = 0.0164, 0.0138), associations between *S-*crotonaldehyde and PM_2.5_, PM_10_, and O_3_ were negative during earlier stages of pregnancy.

## Discussion

In this study we selectively targeted a panel of 15 HSA-Cys^34^ adducts based on previously reported relationships between environmental exposures and oxidative stress (19, 20, 47, 50). Overall, two general trends were identified—(1) exposure to air pollution late in pregnancy (i.e., in the last 30 days), corresponding to the residence time of HSA in plasma, was associated with increased oxidative stress, and (2) exposure to air pollution earlier in pregnancy (i.e., not in the last 30 days) was associated with decreased oxidative stress around the time of birth. These two general trends are discussed below and summarized in Table 3.

### Air Pollution Exposure in the Last 30 Days of Pregnancy

Air pollution exposure during the last 30 days of pregnancy was associated with decreased *S-*sulfinic acid, decreased *S-*γ-GluCys, and increased *S-*crotonaldehyde, which are all consistent with increased levels oxidative stress. Because HSA has a residence time of about a month (40–42), HSA-Cys^34^ adducts measured in newborn DBS samples (collected a day or two after birth) reflect an integration of circulating reactive compounds in the blood during this last 30 days of pregnancy. Therefore, adducts associated with oxidative stress triggered by air pollution exposure late in pregnancy can be directly captured in the newborn adductome.

### Direct Oxidation Products

Exposure to O_3_ during the last 30 days of pregnancy was associated with decreases in *S-*sulfinic acid. While it seems counterintuitive that HSA-Cys^34^ oxidation products would decrease with oxidative stress (i.e., *S-*sulfinic acid), these finding are consistent with previous reports of decreases in *S-*sulfinic acid in association with cigarette smoking (18), chronic obstructive pulmonary disease (19), and ischemic heart disease (19). In addition, decreases in other direct oxidation products (i.e., *S*-sulfonic acid) have been associated with O_3_ exposure (19), which is consistent with our findings. It has been proposed that negative relationships between HSA-Cys^34^ oxidation products and increased oxidative stress may be due to perturbations to the redox proteome and/or are the result of different rates of adduction depending on the oxidation status of HSA-Cys^34^ (e.g., oxidized vs. reduced HSA) (18, 60–62).

### Small Thiol Compounds

O_3_ exposure in the last 30 days of pregnancy was negatively associated with *S-*γ-GluCys. *S-*γ-GluCys is a two-amino acid precursor of GSH, which contains a free thiol that binds to HSA-Cys^34^ forming a mixed disulfide. Oxidative stress can result in the depletion of small thiol compounds in plasma, and therefore decreased levels of *S-*γ-GluCys is consistent with increased oxidative stress caused by exposure to O_3_ in late pregnancy (63).

### Reactive Aldehydes

Finally, Exposure to O_3_ during the last 30 days of pregnancy was associated with increases in levels of *S-*crotonaldehyde. Since *S-*crotonaldehyde is a reactive aldehyde produced through lipid peroxidation, increased levels of *S-*crotonaldehyde are indicative of increased levels of oxidative stress. These findings are consistent with previous findings, where biomarkers of lipid peroxidation were positively associated with O_3_ exposure (64).

### Air Pollution Exposure Earlier in Pregnancy

In contrast with air pollution exposures occurring during the last 30 days of pregnancy, we observed an opposite trend with air pollution exposure in earlier stages of pregnancy (i.e., not in the last 30 days of pregnancy).

### Direct Oxidation Products

*S-*sulfinic acid showed an opposite trend earlier in pregnancy with a negative associated with O_3_. While a positive association was observed between NO_2_ exposure and *S-*sulfinic acid in the 3rd trimester, this association was not significant in the last 30 days. Therefore, we infer that NO_2_ exposure earlier in the 3rd trimester (i.e., not in the last 30 days) is most likely responsible for this trend, which is consistent with our O_3_ findings.

### Reactive Aldehydes

Exposures to PM_2.5_, PM_10_, and O_3_ in the 1st and 2nd trimesters were consistently negatively associated with *S-*crotonaldehyde, suggesting that exposures to air pollution earlier in pregnancy are associated with a decrease in lipid peroxidation around the time of birth.

The residence time of HSA in blood of ~1 month is important to the interpretation of these findings. Adducts measured in newborn DBS samples capture an integration of adducts formed through reactions with circulating small molecules in the infant's blood during the last 30 days of pregnancy. As a result, the newborn DBS adductome does not directly capture adducts related to environmental exposures or biological responses to exposures earlier in pregnancy, but rather likely reflect adaptive mechanisms that may have been primed by air pollution exposures earlier in fetal development. In a previous study, Tissot van Patot et al. (65) observed that infants born at high altitude appeared to develop protective mechanisms against oxidative stress compared with infants born at sea level. In that study, infants born at sea level displayed evidence of oxidative stress during labor, while infants born at high altitude had minimal or no oxidative stress at the time of delivery. The authors hypothesized that fetuses developing at high altitude exposed to chronic levels of oxidative stress during pregnancy developed compensatory responses to oxidative stress by the time of delivery. Along these same lines, we speculate that infants exposed to oxidative stress from air pollution during pregnancy may develop similar mechanisms for combating oxidative stress around the time of birth. If this is true, decreases in oxidative stress-related adducts measured around the time of birth may be due to a heightened antioxidant response primed by earlier exposures to air pollution.

It is well-established that exposures to air pollution during pregnancy causes oxidative stress (9, 15, 66). Because HSA-Cys^34^ adducts measured in newborn DBS samples provide a record of small reactive molecules circulating in the infant's blood during the last month of pregnancy, it is not surprising that exposure to air pollution in the last 30 days of pregnancy was associated with increased levels of oxidative stress captured in the newborn DBS adductome. Adducts that were associated with air pollution in the last 30 days of pregnancy were products of lipid peroxidation (*S-*crotonaldehyde), ROS (*S-*sulfinic acid), and small thiol compounds that provide antioxidant defense (*S-*γ-GluCys). Thus, our adductomics approach captures three distinct layers of oxidative stress using a single assay. Because directly obtaining biospecimens from newborns before and around the time of delivery is rarely possible, measuring protein adducts in newborn DBS samples provides new opportunities to assess prenatal environmental exposures and biological responses to chemical stressors prior to birth.

We also observed an opposite trend with air pollution exposures occurring earlier in pregnancy (i.e., not in the last 30 days), when adducts related to oxidative stress were consistently found to be inversely related to air pollution exposure. We are not suggesting that exposures to air pollution earlier in pregnancy is beneficial to early development. In fact, mounting evidence supports a strong link between exposure to air pollution during all stages of pregnancy and adverse children's health. Rather, we speculate that chronic exposures to air pollution may trigger adaptive changes in redox biology during critical stages of fetal development that may help to shield the infant from environmental stressors around the time of birth.

## Conclusions

Strengths of this study include the innovative approach to assessing associations of air pollution on oxidative stress in an administrative data set with high quality electronic medical record. Although the samples were archived for over a decade, relevant Cys34 adducts and associations with air pollutants were identified. The approach has potential application to other exposures, to other features of neonatal biology, and to development of disease during childhood. Potential confounders of the relationship between air pollutants studied and these neonatal markers at birth are not well-characterized. Although exposure assessment has been validated across the dense monitoring network in southern California (56), exposure misclassification within the single study community and year designed to enhance exposure contrast and to reduce unmeasured spatial confounders may have resulted in misclassification bias. This bias would have been likely to result in attenuated associations with the neonatal outcomes, with some exceptions. Multiple comparisons may have resulted in false positive associations. However, because the biological markers were correlated and represented overlapping pathways reflecting oxidative stress, the results were likely not independent, and we did not adjust for false discovery of positive associations. Because the sample size was relatively small (*N* = 120), power was limited to identify weak causal associations.

In summary, results suggest late pregnancy exposures to regulated air pollutants result in increased neonatal oxidative stress at birth. Exposures occurring early in pregnancy may also alter biology and leave measurable impacts on the developing infant. Further studies are needed to confirm these novel findings.

## Data Availability Statement

The original contributions presented in the study are included in the article/Supplementary Material, further inquiries can be directed to the corresponding author/s.

## Ethics Statement

The studies involving human participants were reviewed and approved by Kaiser Permanente Southern California (KPSC) Institutional Review Board (IRB) reviewed and approved your new study (IRB# 12075). Written informed consent to participate in this study was provided by the participants' legal guardian/next of kin. Written informed consent was obtained from the individual(s), and minor(s)' legal guardian/next of kin, for the publication of any potentially identifiable images or data included in this article.

## Author Contributions

WF, AX, and RM: conception, design, and study supervision. WF, NM, FL, SE, and AX: development of methodology. WF, NM, JC, FL, and AX: acquisition of data. WF, NM, YB, TC, MM, FL, SE, RM, and AX: analysis and interpretation of data, writing, review, and revision of the manuscript. All authors contributed to the article and approved the submitted version.

## Funding

This work was supported by Grants R56ES02812101, 1R01ES02996301A1, P30ES007048, and 1R21ES02677601 from the National Institute of Environmental Health Sciences.

## Conflict of Interest

FL was employed by company Sonoma Technology, Inc. The remaining authors declare that the research was conducted in the absence of any commercial or financial relationships that could be construed as a potential conflict of interest.

## Publisher's Note

All claims expressed in this article are solely those of the authors and do not necessarily represent those of their affiliated organizations, or those of the publisher, the editors and the reviewers. Any product that may be evaluated in this article, or claim that may be made by its manufacturer, is not guaranteed or endorsed by the publisher.
