# Supplementary material for: Human Serum Albumin Cys34 Adducts in Newborn Dried Blood Spots: Associations With Air Pollution Exposure During Pregnancy
Source: Front Public Health. 2021 Dec 23;9:730369. doi: 10.3389/fpubh.2021.730369 (PMC8733257; doi:10.3389/fpubh.2021.730369)
Supplement: Supplementary file 1 [file Table_1.docx]

**Supplemental Table1.** Air Pollutants on log-transformed Adducts, adjusted for gestational age at delivery (in weeks) and child's gender, N=120.

| **HSA-Cys^34^ Adduct** | **Cys^34^ 🡪 Gly** | | | **Unmodified T3** | | **methylation (not Cys^34^)** | |
| --- | --- | --- | --- | --- | --- | --- | --- |
|  | β (SE) | P-value | β (SE) | | P-value | β (SE) | P-value |
| **PM_10_** |  |  |  | |  |  |  |
| Trimester 1 | -0.0001 (0.0036) | 0.967 | -0.0011 (0.0053) | | 0.838 | 0.0057 (0.0052) | 0.281 |
| Trimester 2 | 0.004 (0.0032) | 0.214 | 0.002 (0.0047) | | 0.670 | 0.0066 (0.0046) | 0.154 |
| Trimester 3 | -0.0003 (0.0025) | 0.897 | -0.0001 (0.0037) | | 0.980 | 0.003 (0.0037) | 0.407 |
| Last 30 days in pregnancy | -0.0001 (0.0022) | 0.953 | 0.001 (0.0033) | | 0.769 | 0.001 (0.0033) | 0.766 |
| **PM_2.5_** |  |  |  | |  |  |  |
| Trimester 1 | 0.0035 (0.0107) | 0.747 | -0.0005 (0.0159) | | 0.976 | 0.0178 (0.0156) | 0.255 |
| Trimester 2 | 0.0115 (0.0125) | 0.361 | 0.0053 (0.0186) | | 0.775 | 0.0276 (0.0181) | 0.130 |
| Trimester 3 | 0.0017 (0.0078) | 0.827 | -0.015 (0.0115) | | 0.195 | 0.0034 (0.0114) | 0.764 |
| Last 30 days in pregnancy | 0.002 (0.0053) | 0.709 | -0.0047 (0.0079) | | 0.551 | 0.0044 (0.0078) | 0.574 |
| **O_3_** |  |  |  | |  |  |  |
| Trimester 1 | 0.0039 (0.0037) | 0.301 | -0.0017 (0.0055) | | 0.756 | 0.0027 (0.0054) | 0.623 |
| Trimester 2 | 0.0028 (0.003) | 0.340 | -0.0009 (0.0044) | | 0.831 | 0.0026 (0.0043) | 0.543 |
| Trimester 3 | -0.0038 (0.0037) | 0.302 | 0.0006 (0.0055) | | 0.912 | -0.0067 (0.0054) | 0.221 |
| Last 30 days in pregnancy | -0.0044 (0.0028) | 0.116 | -0.001 (0.0042) | | 0.820 | -0.0066 (0.0041) | 0.106 |
| **NO_2_** |  |  |  | |  |  |  |
| Trimester 1 | -0.0111 (0.016) | 0.490 | -0.0042 (0.0237) | | 0.858 | 0.0014 (0.0234) | 0.952 |
| Trimester 2 | 0.005 (0.0163) | 0.761 | 0.0088 (0.0242) | | 0.718 | 0.0164 (0.0238) | 0.492 |
| Trimester 3 | 0.0122 (0.0133) | 0.362 | -0.0132 (0.0198) | | 0.507 | 0.0093 (0.0195) | 0.634 |
| Last 30 days in pregnancy | 0.0129 (0.0109) | 0.236 | -0.0075 (0.0162) | | 0.643 | 0.0072 (0.0159) | 0.653 |

| **HSA-Cys^34^ Adduct** | ***S*-sulfinic acid** | | | ***S*-methylthiolation** | | ***S*-sulfonic acid** | |
| --- | --- | --- | --- | --- | --- | --- | --- |
|  | β (SE) | P-value | β (SE) | | P-value | β (SE) | P-value |
| **PM_10_** |  |  |  | |  |  |  |
| Trimester 1 | 0.0021 (0.0018) | 0.256 | 0.0057 (0.0052) | | 0.281 | 0.0012 (0.0031) | 0.693 |
| Trimester 2 | 0.0032 (0.0016) | **0.048** | 0.0066 (0.0046) | | 0.154 | -0.0014 (0.0028) | 0.623 |
| Trimester 3 | 0.0001 (0.0013) | 0.962 | 0.003 (0.0037) | | 0.407 | -0.001 (0.0022) | 0.655 |
| Last 30 days in pregnancy | -0.0003 (0.0011) | 0.765 | 0.001 (0.0033) | | 0.766 | -0.0006 (0.0019) | 0.744 |
| **PM_2.5_** |  |  |  | |  |  |  |
| Trimester 1 | 0.0052 (0.0053) | 0.332 | 0.0178 (0.0156) | | 0.255 | 0.0013 (0.0093) | 0.890 |
| Trimester 2 | 0.0107 (0.0062) | 0.087 | 0.0276 (0.0181) | | 0.130 | -0.0008 (0.0108) | 0.939 |
| Trimester 3 | 0.0042 (0.0039) | 0.284 | 0.0034 (0.0114) | | 0.764 | 0.0014 (0.0068) | 0.837 |
| Last 30 days in pregnancy | 0.0034 (0.0027) | 0.200 | 0.0044 (0.0078) | | 0.574 | -0.0001 (0.0046) | 0.985 |
| **O_3_** |  |  |  | |  |  |  |
| Trimester 1 | 0.0043 (0.0018) | **0.021** | 0.0027 (0.0054) | | 0.623 | 0.002 (0.0032) | 0.531 |
| Trimester 2 | 0.0016 (0.0015) | 0.272 | 0.0026 (0.0043) | | 0.543 | -0.0042 (0.0025) | 0.100 |
| Trimester 3 | -0.0049 (0.0018) | **0.0074** | -0.0067 (0.0054) | | 0.221 | -0.003 (0.0032) | 0.352 |
| Last 30 days in pregnancy | -0.0037 (0.0014) | **0.008** | -0.0066 (0.0041) | | 0.106 | 0.0001 (0.0024) | 0.964 |
| **NO_2_** |  |  |  | |  |  |  |
| Trimester 1 | 0.0021 (0.008) | 0.791 | 0.0014 (0.0234) | | 0.952 | 0.0086 (0.0138) | 0.535 |
| Trimester 2 | 0.0145 (0.0081) | 0.075 | 0.0164 (0.0238) | | 0.492 | 0.01 (0.0141) | 0.480 |
| Trimester 3 | 0.0146 (0.0065) | **0.028** | 0.0093 (0.0195) | | 0.634 | 0.0007 (0.0115) | 0.954 |
| Last 30 days in pregnancy | 0.0101 (0.0054) | 0.063 | 0.0072 (0.0159) | | 0.653 | -0.0039 (0.0094) | 0.678 |

| **HSA-Cys^34^ Adduct** | ***S*-methylisocyanate** | | | ***S*-crotonaldehyde** | | ***S*-mercaptoacetamide*** | |
| --- | --- | --- | --- | --- | --- | --- | --- |
|  | β (SE) | P-value | β (SE) | | P-value | β (SE) | P-value |
| **PM_10_** |  |  |  | |  |  |  |
| Trimester 1 | -0.0072 (0.0052) | 0.174 | -0.0088 (0.0039) | | **0.025** | 0.0016 (0.0068) | 0.814 |
| Trimester 2 | -0.0052 (0.0047) | 0.264 | -0.0079 (0.0034) | | **0.024** | -0.0033 (0.006) | 0.586 |
| Trimester 3 | -0.0051 (0.0036) | 0.166 | -0.0049 (0.0027) | | 0.072 | -0.0024 (0.0047) | 0.605 |
| Last 30 days in pregnancy | -0.003 (0.0033) | 0.357 | 0.0003 (0.0025) | | 0.911 | -0.0044 (0.0042) | 0.289 |
| **PM_2.5_** |  |  |  | |  |  |  |
| Trimester 1 | -0.0218 (0.0156) | 0.163 | -0.0248 (0.0116) | | **0.035** | 0.004 (0.0201) | 0.844 |
| Trimester 2 | -0.0233 (0.0182) | 0.203 | -0.0211 (0.0137) | | 0.125 | -0.0096 (0.0235) | 0.685 |
| Trimester 3 | -0.0218 (0.0113) | 0.055 | -0.0231 (0.0083) | | **0.0065** | 0.0008 (0.0147) | 0.956 |
| Last 30 days in pregnancy | -0.013 (0.0077) | 0.095 | -0.0125 (0.0058) | | **0.032** | -0.0029 (0.01) | 0.772 |
| **O_3_** |  |  |  | |  |  |  |
| Trimester 1 | 0.0028 (0.0054) | 0.614 | -0.0089 (0.004) | | **0.029** | 0.0035 (0.007) | 0.617 |
| Trimester 2 | -0.0042 (0.0043) | 0.330 | -0.0097 (0.0032) | | **0.003** | -0.0086 (0.0055) | 0.123 |
| Trimester 3 | 0.0005 (0.0055) | 0.931 | 0.0164 (0.0038) | | **<.0001** | -0.0036 (0.007) | 0.609 |
| Last 30 days in pregnancy | 0.0018 (0.0041) | 0.661 | 0.0138 (0.0028) | | **<.0001** | 0.0028 (0.0053) | 0.599 |
| **NO_2_** |  |  |  | |  |  |  |
| Trimester 1 | -0.0265 (0.0233) | 0.257 | 0.0176 (0.0175) | | 0.317 | 0.0236 (0.0299) | 0.433 |
| Trimester 2 | -0.0015 (0.0239) | 0.949 | -0.0092 (0.0179) | | 0.610 | 0.0294 (0.0305) | 0.336 |
| Trimester 3 | -0.0266 (0.0194) | 0.172 | -0.0337 (0.0144) | | **0.021** | 0.01 (0.025) | 0.691 |
| Last 30 days in pregnancy | -0.0221 (0.0158) | 0.165 | -0.0251 (0.0118) | | **0.036** | -0.0088 (0.0205) | 0.670 |

| **HSA-Cys^34^ Adduct** | ***S*-Cys (-H_2_O)** | | | ***S*-Cys** | | ***S*-hCys** | |
| --- | --- | --- | --- | --- | --- | --- | --- |
|  | β (SE) | P-value | β (SE) | | P-value | β (SE) | P-value |
| **PM_10_** |  |  |  | |  |  |  |
| Trimester 1 | 0.0049 (0.0033) | 0.142 | -0.0031 (0.0047) | | 0.519 | -0.0031 (0.006) | 0.605 |
| Trimester 2 | 0.0026 (0.0029) | 0.377 | -0.0035 (0.0042) | | 0.401 | -0.003 (0.0053) | 0.571 |
| Trimester 3 | 0.0015 (0.0023) | 0.512 | -0.0046 (0.0033) | | 0.162 | -0.0026 (0.0042) | 0.541 |
| Last 30 days in pregnancy | 0.0008 (0.0021) | 0.693 | -0.0047 (0.0029) | | 0.111 | -0.0038 (0.0037) | 0.305 |
| **PM_2.5_** |  |  |  | |  |  |  |
| Trimester 1 | 0.0116 (0.0098) | 0.241 | -0.0071 (0.0141) | | 0.616 | -0.0116 (0.0178) | 0.515 |
| Trimester 2 | 0.0093 (0.0115) | 0.420 | -0.0128 (0.0164) | | 0.437 | -0.0173 (0.0208) | 0.408 |
| Trimester 3 | 0.0053 (0.0072) | 0.464 | -0.0041 (0.0103) | | 0.692 | -0.0057 (0.013) | 0.664 |
| Last 30 days in pregnancy | 0.0008 (0.0049) | 0.879 | -0.0043 (0.007) | | 0.541 | -0.0011 (0.0089) | 0.903 |
| **O_3_** |  |  |  | |  |  |  |
| Trimester 1 | -0.0015 (0.0034) | 0.668 | 0.0041 (0.0049) | | 0.403 | 0.003 (0.0062) | 0.629 |
| Trimester 2 | -0.0034 (0.0027) | 0.221 | -0.0007 (0.0039) | | 0.851 | -0.0007 (0.005) | 0.889 |
| Trimester 3 | -0.0009 (0.0034) | 0.791 | -0.0015 (0.0049) | | 0.754 | -0.0052 (0.0062) | 0.399 |
| Last 30 days in pregnancy | 0.0001 (0.0026) | 0.959 | 0.0002 (0.0037) | | 0.967 | -0.0029 (0.0047) | 0.534 |
| **NO_2_** |  |  |  | |  |  |  |
| Trimester 1 | 0.0185 (0.0147) | 0.209 | -0.0196 (0.021) | | 0.352 | -0.0281 (0.0265) | 0.291 |
| Trimester 2 | 0.0125 (0.015) | 0.409 | -0.0149 (0.0214) | | 0.487 | -0.032 (0.027) | 0.239 |
| Trimester 3 | 0.0106 (0.0123) | 0.389 | -0.0059 (0.0176) | | 0.738 | -0.0108 (0.0222) | 0.628 |
| Last 30 days in pregnancy | 0.0038 (0.0101) | 0.704 | -0.0157 (0.0143) | | 0.275 | -0.0116 (0.0181) | 0.522 |

| **HSA-Cys^34^ Adduct** | ***S*-CysGly** | | | ***S-γ*-GluCys** | | ***S*-GSH** | |
| --- | --- | --- | --- | --- | --- | --- | --- |
|  | β (SE) | P-value | β (SE) | | P-value | β (SE) | P-value |
| **PM_10_** |  |  |  | |  |  |  |
| Trimester 1 | 0.0085 (0.0057) | 0.141 | 0.0049 (0.0069) | | 0.481 | -0.0018 (0.0015) | 0.244 |
| Trimester 2 | 0.0078 (0.0051) | 0.123 | 0.0089 (0.0061) | | 0.145 | -0.0008 (0.0013) | 0.571 |
| Trimester 3 | 0.0054 (0.004) | 0.179 | 0.0055 (0.0048) | | 0.250 | -0.0006 (0.0011) | 0.542 |
| Last 30 days in pregnancy | 0.005 (0.0035) | 0.162 | 0.0023 (0.0043) | | 0.593 | 0.0001 (0.0009) | 0.892 |
| **PM_2.5_** |  |  |  | |  |  |  |
| Trimester 1 | 0.0353 (0.0168) | **0.038** | 0.0294 (0.0203) | | 0.150 | -0.0038 (0.0045) | 0.399 |
| Trimester 2 | 0.0323 (0.0198) | 0.106 | 0.0323 (0.0237) | | 0.176 | -0.0016 (0.0053) | 0.769 |
| Trimester 3 | 0.0161 (0.0124) | 0.196 | 0.0224 (0.0148) | | 0.132 | -0.0047 (0.0033) | 0.153 |
| Last 30 days in pregnancy | 0.0138 (0.0084) | 0.105 | 0.0202 (0.01) | | **0.046** | -0.0024 (0.0022) | 0.282 |
| **O_3_** |  |  |  | |  |  |  |
| Trimester 1 | -0.0035 (0.0059) | 0.554 | 0.0008 (0.0071) | | 0.907 | -0.0023 (0.0016) | 0.137 |
| Trimester 2 | 0.002 (0.0048) | 0.671 | 0.0065 (0.0057) | | 0.249 | -0.0003 (0.0013) | 0.821 |
| Trimester 3 | 0.0004 (0.0059) | 0.945 | -0.0116 (0.007) | | 0.102 | 0.0035 (0.0015) | **0.026** |
| Last 30 days in pregnancy | -0.0007 (0.0045) | 0.871 | -0.0131 (0.0053) | | **0.014** | 0.0018 (0.0012) | 0.129 |
| **NO_2_** |  |  |  | |  |  |  |
| Trimester 1 | 0.0302 (0.0254) | 0.237 | 0.0148 (0.0305) | | 0.628 | 0.0011 (0.0067) | 0.874 |
| Trimester 2 | 0.021 (0.026) | 0.421 | 0.012 (0.0311) | | 0.700 | -0.0006 (0.0069) | 0.927 |
| Trimester 3 | 0.0283 (0.0212) | 0.183 | 0.0444 (0.0251) | | 0.080 | -0.0044 (0.0056) | 0.432 |
| Last 30 days in pregnancy | 0.0205 (0.0173) | 0.238 | 0.0297 (0.0206) | | 0.152 | -0.002 (0.0046) | 0.672 |

**S*-mercaptoacetamide = 0 had been imputted to = 0.0053446
